# A qualitative exploration of active ingredients and mechanisms of action of an online singing programme with mothers experiencing postnatal depression during the COVID-19 pandemic: SHAPER-PNDO study

**DOI:** 10.1186/s40359-024-02213-7

**Published:** 2024-11-30

**Authors:** Alexandra Burton, Rebecca H. Bind, Rachel Davis, Lorna Greenwood, Ching Yin Lee, Carolina Estevao, Katie Hazelgrove, Celeste Miller, Kristi Priestley, Lavinia Rebecchini, Tim Osborn, Hannah Dye, Paola Dazzan, Anthony Woods, Nikki Crane, Carmine M. Pariante, Daisy Fancourt

**Affiliations:** 1 Department of Behavioural Science and Health, University College London, 1-19 Torrington Place, London, WC1E 7HB UK; 2 Department of Psychological Medicine, Institute of Psychiatry, Psychology and Neuroscience, King’s College London, 5 Cutcombe Rd, Brixton, London, SE5 9RT UK; 3 Evidera, The Ark, 201 Talgarth Rd, Hammersmith, London, W6 8BJ UK; 4 Breathe Arts Health Research, The Clarence Centre, 6 St George’s Circus, London, SE1 6FE UK; 5 Culture Team, King’s College London, Somerset House East Wing, Strand, London, WC2R 2LS UK

**Keywords:** Depression postpartum, Postnatal depression, Qualitative research, COVID-19, Creative health, Singing intervention, Online delivery

## Abstract

**Background:**

Social distancing restrictions and the suspension of in-person treatment and support contributed to an increase in postnatal depression during the coronavirus disease 2019 (COVID-19) pandemic. Creative health interventions can help to alleviate anxiety and depression, with studies showing that singing is particularly effective for supporting the mental health of new mothers. We adapted an in-person group singing programme (Breathe Melodies for Mums (M4M)) to online delivery during the COVID-19 pandemic to support the mental health of new mothers, and, in a feasibility study, found improvements in postnatal depression (PND) symptoms at 6-month follow up. The current qualitative study aimed to explore how and why M4M-online impacted the mental health of those taking part.

**Methods:**

We took a theory-based approach using the INgredients iN ArTs in hEealth (INNATE) Framework of ‘active ingredients’ and the Multi-level Leisure Mechanisms Framework of ‘mechanisms of action’ to identify and categorise intervention components and change mechanisms. Iterative consensus building between three researchers was complemented by qualitative semi-structured online interviews with 24 women experiencing PND symptoms who took part in M4M-online. Data were analysed inductively using reflexive thematic analysis.

**Results:**

Consistency was found between the online and in-person interventions in active ingredients relating to project design, content, programme management and the composition of the group. Key differences were in the social and contextual ingredients. Psychological, social and behavioural mechanisms for improved mental health and wellbeing included: (1) Increased self-confidence as a mother, (2) Increased positive emotional responses, (3) A supported exploration of self-dentity, (4) Reduced loneliness and isolation, (5) Increased social bonding and connections with family and (6) Enhanced sense of time through new routines.

**Conclusions:**

Participating in online group singing can support new mothers experiencing PND by triggering psychological, social and behavioural responses that lead to improved mental health. Key programme features are identified which can be used to design future online creative health interventions or tailor in-person activities for remote delivery to support populations who may face practical and social barriers to attending in-person.

**Supplementary Information:**

The online version contains supplementary material available at 10.1186/s40359-024-02213-7.

## Introduction

Postnatal depression (PND) affects approximately 10–15% of mothers within the first year after childbirth [1]. It is characterized by low mood, anxiety, sleep disruptions, irritability, anhedonia, fatigue, and suicidal thoughts in severe cases [2]. Risk factors for PND include low perceived social support from partners and family, and social isolation during and after pregnancy [3–5]. If left untreated, PND may predispose mothers to poorer psychological well-being, chronic depression, and difficulties in bonding [6, 7], which can disrupt infants’ emotional, cognitive and behavioural adjustment into adolescence [8, 9]. Yet, PND is widely under-reported due to barriers to help-seeking arising from the stigma, shame and guilt associated with mental health disorders [10, 11]. Because of the potentially long-lasting impacts of PND, it is vital that appropriate support and treatment is offered and that the mechanisms underlying effective interventions are understood.

The postnatal period is widely accepted as a “vulnerable time” where mothers are susceptible to changes in social circumstances and thus require higher levels of social support [12]. The coronavirus disease 2019 (COVID-19) pandemic led to an increased PND prevalence in the UK due to unprecedented COVID-19 social distancing restrictions, fear of the virus, and uncertainty about the future [13, 14]. An English population-based maternity survey study found that PND prevalence doubled to 23.9% at the height of the pandemic [3]. The lockdown restrictions also increased anxiety and loneliness experienced by expectant mothers and new mothers alike [15, 16], exacerbating existing PND risk factors such as social isolation and lack of social support. In addition to these changes, many mothers experiencing PND struggled to receive timely treatment due to not being able to access in-person healthcare during the pandemic [16].

There is a growing evidence base demonstrating that engagement in creative activities can improve mental health and wellbeing [17]. Singing has been identified as a particularly powerful tool for supporting adults with mental health problems through promoting increased social bonding, enjoyment and relaxation [18, 19]. Singing has also been found to support the mental health of new mothers by reducing stress and promoting mother-infant bonding [20, 21]. Previously, participation in an evidence-based 10-week community singing group (Breathe Melodies for Mums (M4M)) was associated with significantly faster recovery in women experiencing moderate to severe PND symptoms compared to women attending play groups and non-intervention controls [22]. These changes were attributed to a range of mechanisms with participants reporting an increased ability to calm their babies through singing, an enhanced sense of achievement, rediscovery of self-identity as a new mother, and enhanced mother-infant bonding [23]. Having established clinical effectiveness, M4M is currently being scaled up and tested in the community as part of a large ‘Scaling-up Health Arts’-research programme (SHAPER) [24].

The COVID-19 Pandemic led to the suspension of in-person community activities, with group singing deemed as a high-risk activity due to the greater potential for virus transmission via respiratory droplets [25]. During this time, many in-person arts activities moved online [26] and M4M was adapted into a six-week online intervention which aimed to replicate as closely as possible its face-to-face counterpart [27]. Online delivery enabled participants to be recruited from across the UK and mitigated COVID-19 related obstacles to participating in group activities such as fear of contracting COVID-19 at a time when access to services and support were severely restricted [27, 28]. M4M-online was associated with significant reductions in PND symptoms, anxiety and stress with positive effects observed at weeks three and six that were maintained at 6-month follow-up, however no impact of M4M-online was observed on quantitative measures of social support or loneliness [29].

Having identified the positive impact of M4M-online on mental health outcomes, it is important to understand how and why these effects occurred so that new online interventions can be developed or tailored to meet the needs of this group. Theoretical work on arts interventions and health have conceptualised the component parts of arts interventions as ‘active ingredients’, comprising elements of the activity people engage in and the broader social and contextual environment in which engagement occurs [30]. Online engagement has clear differences in all three of these categories of ingredients. For example, previous research on virtual choirs has shown that participants engaging virtually report lower and more variable social presence than participants of in-person choirs [31]. Yet in the M4M-online intervention, the outcome findings suggested comparable benefits to mental health in the in-person intervention [22, 29]. This raises the question as to whether the ‘core’ ingredients most responsible for effects were nonetheless still present, even if there were changes to other ingredients that may have been more peripheral. Active ingredients can in turn trigger causal processes or “mechanisms of action” that lead to health outcomes. The Multi-Level Leisure Mechanisms Framework derived from a multi-disciplinary review of over 600 potential mechanisms that are triggered by arts and leisure engagement theorised these mechanisms to be biological, social, psychological and behavioural [32]. Again, it is important to understand whether the mechanisms activated for M4M-online differed from the in-person intervention, to inform future adaptations.

## Research aim

To identify the (i) active ingredients and (ii) mechanisms of action of an online singing intervention (M4M-online) that contributed to improvements in mental health and wellbeing among mothers experiencing postnatal depression.

## Methods

**To identify the active ingredients**, we used the INNATE (INgredients iN ArTs in hEalth) framework. INNATE is a theoretical model for identifying the ‘active ingredients’ in arts in health interventions [30]. The concept ‘active ingredients’ has its origins in pharmacological research for describing the elements of a pharmacological intervention responsible for its therapeutic action (i.e. the ‘intervention components’ or ‘interacting components’) [33]. However, the approach has become increasingly prominent in behavioural interventions to map how different mechanisms of action are triggered and to compare similar interventions to understand why some are more effective than others [34, 35]. INNATE provides a bespoke model and checklist for mapping the ingredients in arts in health interventions and is designed to support the design and implementation of interventions, their evaluation and their replication, scalability and sustainability [30]. Ingredients are categorised as relating to the *project* or content of the activity, the *people* including how people interact and who is involved in the activity and the *context* or environment in which the activity is delivered. AB, DF and LG completed a comprehensive mapping of the key project, people and contextual details for both the M4M-online and previous M4M in-person programme [22], using an iterative approach to the identification of each ingredient until full consensus on each ingredient was achieved.

**To identify the mechanisms of action** (the processes that causally relate ingredients to intervention outcomes [32]), individual semi-structured qualitative interviews were conducted with M4M-online participants after they had completed the singing programme.

### The intervention

M4M-online consisted of six weekly one hour online singing sessions with 3–7 participants in each group. The groups were held via Zoom and were facilitated by a professional singing lead. The intervention was reduced to 6-weeks rather than the 10-weeks offered in person to reduce the likelihood of participants disengaging and because impacts on PND symptoms were identified from 6-weeks onwards in the original in-person trial [22]. Breathe Melodies for Mums is tailored specially towards mothers experiencing PND as all participants have shared lived experience of PND, and like with in-person delivery, uncomplicated and easy to remember songs and lyrics were used to support participants who may be struggling with symptoms of PND such as poor concentration, memory, sleep disturbance or low confidence. Lyrics to songs were posted in the chat function to support participants when their PND symptoms may be making it difficult to follow and, like in the in-person delivery, songs were chosen from a range of different cultures / languages to promote inclusivity. The sessions were supported by a Breathe Arts Health Research co-facilitator who assisted with technological support and troubleshooted any in-session issues, as well as being the main point of contact for participants from recruitment to completion of the programme with responsibility for safeguarding. Participants sang from their homes while being muted at certain times from each other but were able to hear and follow the singing lead throughout. The singing lead also used pre-recorded backing tracks containing vocals in multiple parts and harmonies to support delivery and amplify the experience of singing with others. Participants were signposted to a WhatsApp group to connect with one another outside of the singing sessions and given unstructured time at the beginning and end of each session to talk to the singing lead, co-facilitator and/or other participants.

### Participants

Participants were eligible for the trial if they were aged 18 years or older, were able to understand English, had internet access, capacity to consent, a child between 0 and 9 months old and reported PND symptoms defined as a minimum score of 10 on the Edinburgh Postnatal Depression Scale (EPDS) [36]. Participants were recruited to the trial via different routes including social media, posters and leaflets displayed in community centres, signposting via health and social care professionals and a local National Childbirth Trust (NCT) group. For this qualitative study, all participants who attended one or more online singing sessions as part of the trial and who had consented to take part in an interview were invited to participate.

### Procedures

The study was approved by the King’s College London (KCL) Research Ethics Office (KCL Ethics Reference: HR-20/21–19659). Participants gave their written consent to be contacted for the interview when consenting to take part in the wider feasibility study. The study interviews were conducted between April and October 2021. Participants were invited to take part via email by AB up to two weeks after attending their final session. Most interviews were conducted between two and four weeks after the final session, however one participant had to reschedule multiple times and was interviewed eight weeks after the final session. Interviews were conducted by AB, a female senior research fellow with expertise in conducting qualitative research with people experiencing mental health problems and on the impact of the COVID-19 pandemic on mental health. All participants were given the opportunity to ask questions in the initial email exchange and again before the interview commenced. Interviews followed a semi-structured topic guide (see Supplementary Material) developed by AB, DF, and RD to explore (i) experiences, highlights, and challenges of taking part in the sessions, and (ii) how and why, if at all, the singing groups impacted mental health, wellbeing, thoughts, and behaviours around motherhood. Interviews took place remotely via video call and were audio-recorded. Interviews lasted between 22 and 56 min (average 40 min). Audio-recordings were transcribed verbatim by an external company (TP Transcription) and any identifiable information such as names or locations were subsequently removed from the transcripts. Transcripts and recordings were stored within the University College London (UCL) Data Safe Haven, a certified secure data storage system.

### Data analysis

Interview data were analysed inductively using reflexive thematic analysis [37, 38]. Our research methodology was informed by critical realist ontology whereby, we sought to understand mother’s views of participating in online singing groups and how the groups supported them through in-depth exploration of their personal accounts, stories and experiences. Transcripts were imported into NVivo 12 software to enable data management and coding. DF and AB independently coded three transcripts and met to discuss emerging mechanisms of action as well as active ingredients of the singing intervention that triggered general points of interest that aligned with the research aim. AB then coded all remaining transcripts using language and words closely aligned with participant accounts. Codes were then grouped into similar topics and used to inform the development of themes. A preliminary list of themes was shared and discussed with DF and RD for comment, interpretation, and reflection before the themes were finalised. Themes were then categorised as either psychological, social, or behavioural as defined by the Multi-Level Leisure Mechanisms Framework [32].

## Results

### Active ingredients

**Fig. 1:**
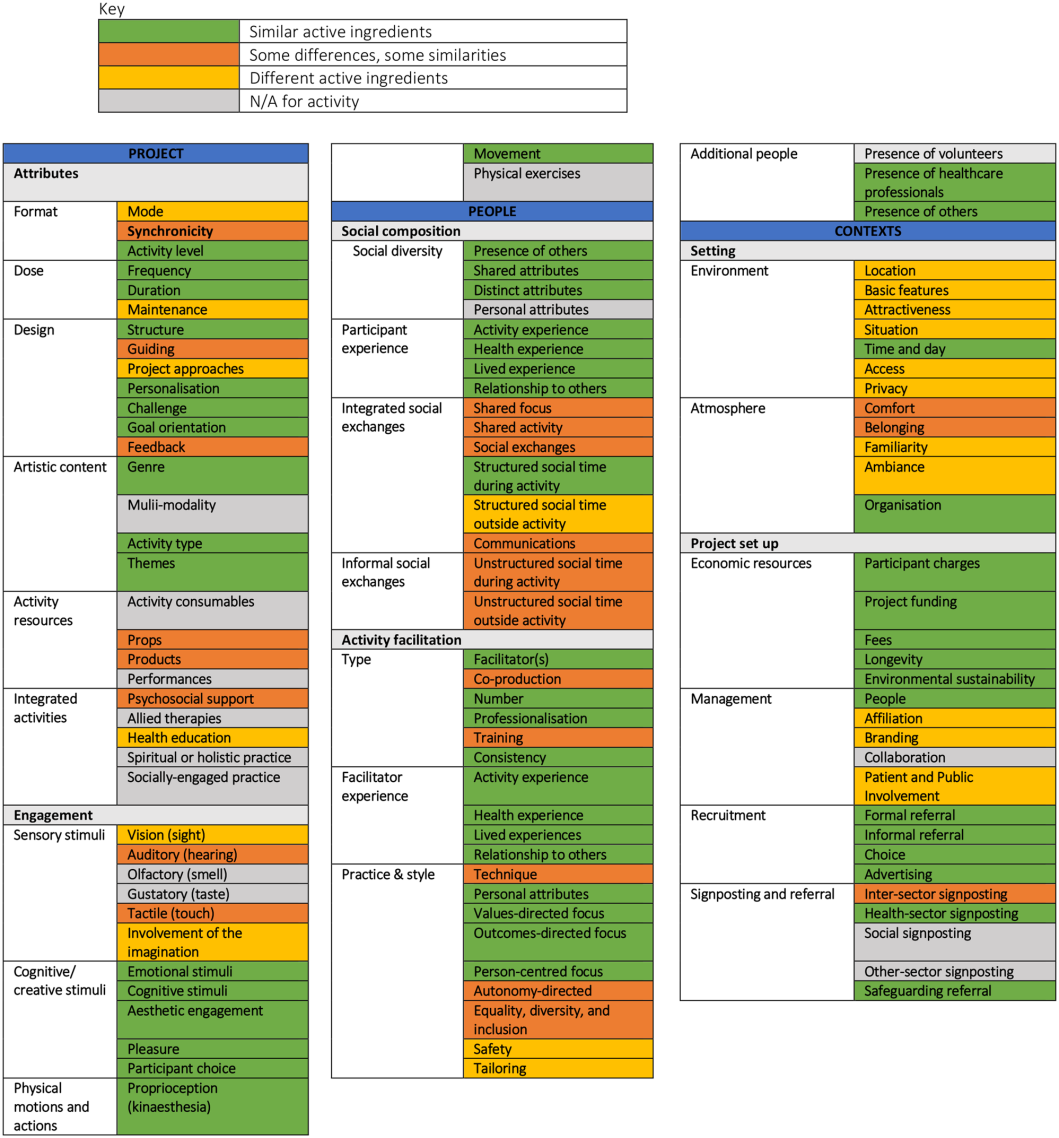
Colour chart comparing ingredients of M4M-online vs. M4M in-person

Figure 1 shows the full list of M4M programme ingredients identified by the research team (AB, DF and LG) presented in the INNATE framework and colour-coded according to the similarities between M4M-online and M4M-in-person. The full checklist of ingredients using the comprehensive INNATE worksheet is provided in the Supplementary Material.

In all, there were several key similarities in the project (green), showing shared ingredients. However, other ingredients differed slightly in the online version (orange), while others were very different (yellow).

### Project

In the mapping exercise, several project ingredients were identified as being the same across the online and in-person programmes including the frequency (weekly) and duration of sessions (one-hour), many of the design features, the artistic, emotional and cognitive content and breathing and stretching warm-up exercises. Project ingredients that shared some similarities included singing together in real time (albeit predominantly on mute in the online programme) and the facilitator sometimes playing a musical instrument (although the use of other props varied as children’s toys, cushions, blankets and mats were supplied in-person however participants in the online group provided their own materials and props including homemade instruments, muslin cloths and scarfs).

Key differences included the number of weeks the programme ran for (M4M-online was a shorter 6-week programme compared to 10 weeks for the in-person programme), the approach to learning the songs, with those online predominantly singing on mute with accompanying backing tracks compared to participant’s directly responding to the singing lead and singing together through harmonies and rounds during the in-person programme and the presentation of song lyrics in the chat function online as opposed to having no access to printed lyrics during the in-person programme.

### People

Participants were adult mothers of children aged nine months or younger at the start of the programme and experiencing symptoms of postnatal depression. No previous experience of arts activities in healthcare contexts was required to participate but several participants had previous experience of singing in choirs, while others had no formal group singing experience. Mothers engaged in the programmes with their babies and group sizes were similar across both formats (between 3 and 12 participants online vs. 3–15 in-person). The programme staff were also similar across both formats; the groups were led by a consistent professional singer with experience of leading similar workshops with mothers and babies and co-facilitated by a member of Breath Arts Health Research who provided practical support to both the singing lead and participants. The co-facilitators were unable to provide hands on support to mothers and their babies during the online programme, however they supported participants to overcome barriers to joining the sessions such as sending weekly text reminders, following up any participants who were absent from a session to understand what might help them to attend and providing signposting to other support services.

Social interactions were similar across both formats in that participants jointly focused on the singing lead to learn the songs and participants were given time to chat at the beginning and end of the sessions. In the online sessions however, participants could also watch themselves on the screen with their babies. The biggest difference was that participants in the online programme had less opportunity for unstructured face-to-face social time outside of the activity and many were unable to meet in person locally outside of the group sessions because the groups were offered to people across England. They were however signposted to a WhatsApp group and could arrange to meet up with those who were local to them and when pandemic restrictions allowed. Those attending the in-person groups would often go for a coffee together after the sessions or proactively arrange to meet up outside of the sessions.

### Context

The main contextual similarities between the online and in-person programmes were the economic resources and management needed to run each programme including sessions being free to attend, “behind the scenes” administrative support, sessions taking place between 10am and 4pm on weekdays and the same range of referral routes and programme advertising including social media, word-of-mouth and formal recommendation from healthcare professionals.

The key difference between M4M-online and the in-person programme occurred in the contexts in which they were delivered. The online space afforded a completely different environment (via Zoom on laptops and mobile phones within the participant’s home) compared to the in-person programme, where participants travelled to Children & Family centres and sang together in a private physical space. While the characteristics of the spaces were different, there were shared elements of comfort, belonging and a welcoming atmosphere created by the different environments. While singing together was an aim of both the online and in-person groups to foster a collaborative and inclusive environment, many participants in the online group reported that singing together off mute did not work well via the online platform.

### Participant characteristics

35 of the 37 women who took part in the main trial consented to be contacted for the qualitative interview, of whom 24 women participated. Of the 11 women who did not take part in an interview, ten did not reply to the invitation to participate and one withdrew from the main trial prior to six-week follow up.

Participants were aged 29–44, predominantly White, married, employed/on maternity leave and highly educated. Half of the sample reported a diagnosed mental health problem including anxiety, eating disorders, and mood disorders and just under half had a long-term physical health condition including respiratory, endocrine, cerebrovascular and gynaecological disorders. Singing intervention attendance was high with an average of 5 out of a possible 6 sessions attended. Characteristics of the 24 women who took part are presented in Table 1.

**Table 1:** Participant characteristics

Characteristic	*N* = 24 (%)
Age (Mean, range)	36 (29–44)
Ethnicity	
White	20 (83%)
Indian	2 (8%)
Mixed ethnicity	1 (4%)
Other ethnicity	1 (4%)
English as first language	21 (88%)
Marital/living status	
Married/cohabiting	23 (96%)
Single (no partner)	1 (4%)
Educational qualifications	
Higher education (degree/diploma)	23 (96%)
GCSEs/O levels	1 (4%)
Employment status	
Employed/maternity leave	21 (88%)
Unemployed/student	3 (13%)
Diagnosed health conditions	
Mental health condition	12 (50%)
Physical health condition	11 (46%)
Number of sessions attended (Mean, range)	5 (1–6)

### Mechanisms of action

We identified six key psychological, social and behavioural mechanisms by which M4M-online positively impacted the mental health and wellbeing of participants. Psychological mechanisms included (i) increased self-confidence as a mother, (ii) increased experience of positive emotional responses and (iii) a supported exploration of self-identity. Social mechanisms were (iv) reduced loneliness and isolation and (v) increased social bonding and connection with family. One behavioural mechanism was identified: (vi) enhancing a sense of time through providing new routines. These mechanisms are described alongside the active ingredients responsible for each mechanism with accompanying quotations from participants below. All participant names have been replaced with an anonymised ID number after each quote.

### Psychological mechanisms

#### Increased self-confidence as a mother

Participants described being given *“a toolkit of things that I can use”* to help them calm and soothe their baby before bedtime, to energise and engage during playtime and to distract during nappy changing, feeding or during times when they were unable to fully engage with their baby due to competing household activities. This was achieved through learning a range of songs with different styles and tempos that could be harnessed in different situations. This enabled participants to build their psychological strength and feel more in control of their baby’s behaviour which in turn increased their confidence in parenting, improving their own mood and ability to cope:

I think that you’re very delicate and you’re probably at your most fragile after you’ve had a baby both in terms of physical health and mental health, and it’s easy to not have confidence. I think that I suffered from a lack of confidence and an inability to know what to do when my baby was upset and it just sort of stimulated this memory that actually sing, you know, you can sing, you know songs, so just sing! And it works (P04).

Observing the positive impact that singing had on their baby whilst participating in the group sessions also helped participants to feel more confident in singing to their baby both within the home but also in front of other people, which helped them to feel more in control of their baby’s mood and increased their confidence when taking their child out in public:

It’s increased my repertoire and confidence…and if I’m out and about sometimes I’m singing away to her, and you know sometimes I’ll scat to < baby > which is funny. Yes, so it’s given me that confidence just to be like, do you know what I don’t care because she enjoys it and it calms her as well, it does calm < baby> (O21).

Many participants also described being able to access the singing group in an online format during the pandemic as a catalyst for feeling more confident in seeking out opportunities for in-person social interactions and activities once COVID-19 pandemic restrictions were lifted. The welcoming and safe environment created by the artists and facilitators delivering the programme contributed to this:

I think it made me braver about going to do things with people I don’t know, specifically mother and baby classes. As we said, like it was a safe space and it was all really fun and really worthwhile, so actually being able to do that as a starter has meant that I’ve gone to other baby classes and not worried about not knowing anybody and going there for the first time. I think it was the stepping stone to feeling like I could achieve things as a mum and doing things like that with my son (P04).

#### Increased experience of positive emotional responses

Participants attributed a range of positive emotions to the online singing programme and reported feeling energised and euphoric both during and after the sessions. Participants described their anticipation and excitement to take part in future sessions and reported being able to return to the songs they had learned in their own time to help improve their mood:

If I’m feeling a bit low- I don’t know, just any time of day, I’ll put on sound cloud and we’ll sing away to Boats Go Home or one of them, and it will just brighten- I think it’s like an endorphin hit isn’t it, singing. It’s like exercise and stuff like that. Eating chocolate. So, it just gives you a hit of just feeling nice (R07).

Many participants described feelings of joy whilst singing together as a group and described the sessions as enjoyable, playful, and fun. This was credited primarily to the facilitator creating a safe, informal and relaxed space where people did not feel judged by their singing ability, incorporating a mix of popular songs and new songs in different languages and changing lyrics to well-known songs to incorporate humour into the sessions:

The sessions were just fun whether it was singing as a group or < Name of facilitator > often made us sing a line on our own and it was really nice that we were in a group of ladies that were all really confident in doing that or not worried about doing that because it was a safe space. And as I said to you my singing skills you know I’m almost deaf but I love singing and after a couple of weeks I didn’t even think twice about doing that (N17).

As well as feeling uplifted by singing, some participants described using singing within and outside of the sessions to release negative emotions and as a tool to help them relax:

It’s definitely taught me to, because singing relaxes me as well so singing every day has allowed me to relax more so that then opens up many more things that we could be doing. So I’m not getting so het up with like, “must do this, must do this”. And then all we need to do is just sit back and just, “okay what’s going on”. Evaluate and then move forward (O21).

#### A supported exploration of self-identity

Most participants described previously engaging with music, singing or choirs before the birth of their child as a motivating factor for taking part in the singing groups. For some this was an opportunity to reconnect with their identity as a singer, re-engage with their love of music or revisit positive memories triggered by familiar songs:

Once I did Melodies for Mums, I found myself going back through my favourite music, and then singing to her. I still do that now, which is fun for me, because I do really like music, and obviously she enjoys it as well (R01).

Having time and space to engage in something enjoyable for themselves, rather than just participating in an activity for their baby was experienced as an important element of the programme because it enabled participants to reconnect with an identity outside of motherhood and contributed to a reduced loss of self:

Just having space that’s for me, where I’m not just mum for five minutes. Because I think that’s one of the things becoming a mum, it’s actually really lonely. And I found this with my daughter a bit, but you just lose yourself, and you feel like you don’t exist anymore, and you’re just mum. So having something where it was like, yes, it’s a mums’ singing group. But it’s not all about baby. Having that head space just for a bit of fun for me, baby will be there, he doesn’t have to be there, it’s fine (N14).

This led some participants to realise that self-care and engaging in activities without their baby going forward was important for their mental health and for self-restoration:

It’s reminded me that I should prioritise that as, possibly not with the baby, but as something I do for myself, that that is something I might enjoy. Because I think it’s been so long now. It’s hard to remember sometimes the things that you like to do, that haven’t been a possibility for so long (P19).

### Social mechanisms

#### Reduced loneliness and isolation

Participants experienced the groups as friendly, supportive and some described feeling part of *“a community through singing together”*. Being in a small group and being able to chat in between singing together and at the end of the sessions helped participants to build connections with one another:

I think the fact it was small groups was really nice because they’d stop and chat part way through and things like that, you know, if there’d been 200 people and you just logged in and it was anonymous– it’s not like we logged in and talked about our feelings, no, < facilitator > would just do a bit of chat, like how are you today, it was just small talk chatting, it wasn’t sort of how are you feeling today, you know, like that, but it was– yes, it was just nice and easy (P12).

Some participants found it helpful to actively share the challenges associated with parenthood, as well as their experiences of emotional difficulties with others in the group. Sharing these difficulties in turn made them feel validated and less alone:

It was definitely nice to hear other mums say or feel similar things to myself. So, that definitely impacted the way I felt because I knew that, okay, I’m not a terrible mum because my son cries sometimes because this lady also has those feelings, she’s not a terrible mum (N09).

Others felt less isolated just by knowing that other mums were experiencing low mood or anxiety without having to discuss how they were feeling. This shared but unspoken experience was described as *“comforting”*,* “healing”* and made participants feel *“normal”*:

I think just connection. It’s helped me to, like, I guess, deal with perhaps the feelings of isolation, and helped me channel my negative feelings into something a little more positive. It was reassuring to know that other mums were going through similar things, but again, not necessarily having to discuss it (N03).

Receiving positive feedback about their own baby’s behaviour from group members *“people would say his smile has made my day and I’d think*,* oh that’s nice…to feel proud of them”* and observing other mums interacting with their babies also helped participants to feel less alone and normalised their own difficult experiences:

I was still finding it quite difficult if I saw friends or family and she was crying a lot, I still found that quite stressful. And also I saw the other babies basically in the same position, I don’t know whether they had colic… I was kind of like, oh, this is normal, and it made me feel less self-conscious (R01).

While most participants described the benefits of socialising and connecting with other mums during the sessions, most reported difficulties connecting and bonding with one another online and described a preference for attending the group in-person. This was attributed in part to multiple functionalities not being supported by the technology, such as not being able to access the lyrics on screen at the same time as viewing the singing lead alongside other participants. Singing together off mute as a group was also described by some as challenging:

And then when we did come off mute occasionally to try to sing altogether, it was kind of, it was really broken up. So it was nice to do occasionally but it definitely wouldn’t work to do it too often (M22).

Some participants had hoped to make lasting connections and meet one another once the groups had finished, but this had not materialised due to living in different locations. Others felt that more time was needed within the sessions to socialise and bond:

I think, you know < Name of facilitator > was always very good at facilitating a bit of, “Hey how are you and what’s going on?” It’s nice but it’s also a bit difficult as well because there’s not really…a connection with the other people apart from having a baby. I think that’s the difficulty with Zoom is, it’s all right if you have a connection with people in real life beforehand and then you have a web interface, but otherwise it’s quite hard to really get a sense of who people are and whether or not, you want to have further conversations (O12).

#### Increased social bonding and connection with family

Participants experienced a strengthening of bond and connection with their baby through singing. For some participants, singing provided them with the opportunity to feel more present with their baby than they had previously, *“from just sort of waiting for her to fall asleep so that I can do something I want to do…to feeling more like I’m a mum who wants to do things with her child”* which in turn improved their mood:

I was feeling that I attended to his needs but may be mechanically, or doing this while feeling that I wasn’t functioning properly. So, a façade of everything is going well so that the baby isn’t affected but feeling anxious inside. And during or after the sessions I attended to his needs and I enjoyed it, a real façade wasn’t needed anymore it was me being here with him. So, I liked the being in the moment that music provides, and I took this enjoying the moment into the rest of our everyday life (N10).

Some participants also spoke about the impact of the singing group on the whole family. Having access to songs outside of the sessions helped to reinforce singing at home and shared experiences of singing together which strengthened family bonds and made everyday activities more enjoyable:

My daughter, my husband, they’ve all heard me singing enough that they picked up on some of them. And on Saturday we take my daughter swimming, and just as we were loading everything back in the car to leave swimming, she was singing, “I want someone to buy me a pony.” And then my husband was singing it back to her doing the chick chock chick chock. And it was just lovely… It has a lasting impact beyond just those six weeks (Q18).

### Behavioural mechanisms

#### Enhancing a sense of time through providing new routines

Participants described sometimes feeling in a “rut” before they joined the online singing programme, spending long periods of time with their babies at home during the pandemic with little variation in routine or activities. For some, attending the singing group added a new structure to their day:

The support came from having something to do every week. I really had very little structure or scheduled going on because of life so it was, you know I think I like to be busy, and I hadn’t been - well I had been busy but busy purely feeding on a constant basis with a baby that wasn’t feeling very well and so I guess for me it was nice to feel I was doing something a bit for myself as well as for him, and to have that regular time in the day to do it (O12).

Some participants continued to introduce new routines into their day once the singing programme had ended, using the songs they had learned and becoming more creative in their interactions with their babies:

I think it has made me sing much more to them, so that has felt really positive and when you start to sing more to them you feel more like playful with them as well. It’s helped in that sense, feeling a bit more creative because sometimes when you have the whole day stretching out, particularly when I have both of them and I sort of wonder what activities I’m going to do, I think it has helped reignite a bit of creativity when it comes to that (R12).

## Discussion

To our knowledge this is the first qualitative study exploring how a group singing programme (M4M) adapted to run online during the COVID-19 pandemic supported the mental health of women with postnatal depression. The online format was implemented directly in response to the suspension of in-person groups due to social distancing restrictions and provided an opportunity to explore whether similar ingredients and mechanisms to improved mental health might be observed by participating online as in person. Our study helps interpret the findings of the feasibility trial (29) by mapping out programme ingredients that seemed to trigger mechanisms which then impacted PND symptoms, as well as identifying ingredients unique to online delivery such as being able to sing on mute at home which led to increased confidence in singing.

A key feature of the outcome findings previously reported on the M4M-online intervention was the similarity in the reduction in EPDS scores over six weeks to the original M4M-in-person intervention [22, 29]. Notably, this was found despite the mode of delivery (online vs. in person) being quite different. In this current study, by mapping the active ingredients in the trial using a theory-informed approach, we were able to differentiate which ingredients were ‘peripheral’, in that changes in them did not materially affect the ability of the intervention to achieve the primary outcome, vs. which ingredients were potentially ‘core’ for such similarities in the primary outcome to be achieved. The key differences between the formats were the environments in which the programmes were delivered, the atmosphere of a virtual rather than in-person setting, and the dominance of singing on mute, using backing tracks and being unable to hear other participants sing online. These ingredients were largely context-based or related to the technical delivery of the project. This suggests that modification of such elements can occur without diluting the psychological benefits of the programme. That said, participant feedback on the online experience was not always positive. Similar to other studies in populations with chronic health conditions [39], participants acknowledged the ease of taking part from home and the benefits during an unprecedented time of social change. But many expressed a preference for attending M4M in-person if given the option.

We also identified several shared or “core” ingredients across the online and in-person [22] formats which may be key to supporting the mental health of new mothers. This included the frequency and duration of sessions and the artistic and emotional content of the songs and singing activities. Participants had similar levels of previous engagement with group singing across both formats and shared experiences of motherhood and postnatal depression. Previous research has identified that shared experience of health problems among online creative health programme participants are important for engagement and feeling supported [40, 41]. The artists and facilitators delivering the online and in-person programmes shared similar skills and previous experience of delivering community singing programmes. Key contextual similarities were the resources including staffing used to deliver, recruit to and manage the programme and the creation of a safe and inclusive environment within both the online and in-person spaces. It is likely that some or all of these ingredients are core to achieving intervention outcomes. That said, further research would be needed to confirm if modifications to these ingredients could still be made without adversely influencing outcomes.

Our study also demonstrated that M4M-online targeted a range of psychological, social and behavioural mechanisms including increased participant confidence in interacting with their babies, a supported exploration of self-identity, improved social bonding with their babies and families and reduced feelings of loneliness and social isolation. Attending the groups also provided participants with new routines and increased their experience of positive emotional responses. Importantly, these effects were felt both during and outside of the singing sessions as participants implemented what they had learned into their daily lives and routines. These findings help us to understand why participating in M4M-online led to reductions in stress, anxiety and PND symptoms in the feasibility trial [29]. Most of the mechanisms identified in the present study also mirrored those produced by the in-person M4M groups including increased bonding between mothers and their babies, supporting mothers to establish new routines, an enhanced sense of identity and singing as a tool for mothers to calm their babies [23]. These same mechanisms have also been reported in research into other online creative health programmes including increased positive emotional response [40, 42, 43], increased confidence in communicating with others [40] and reduced loneliness and isolation [40, 43, 44]. One study evaluating online group dance sessions for young people with anxiety and depression identified increased feelings of social connectedness to others as key for supporting their mental health [41]. We did however identify an additional mechanism around increased self-confidence as a mother, which was not reported in the original M4M-in-person trial. However, this encompassed being able to calm their babies, which was a mechanism of the original trial, and the mothers in the original trial described an increased sense of achievement, which suggests similar mechanisms.

This study also allowed for an understanding of how the ingredients and mechanisms in the intervention interacted to impact outcomes. While participants in our study qualitatively reported feeling less lonely and socially isolated due to meeting other mothers with PND and being able to share the challenges of motherhood, many expressed a dilution of impact through the online format because participants were geographically spread and unable to meet socially after the sessions (alterations in the active ingredients of informal social exchanges and environment). Many of the participants in our study expressed a preference for in-person sessions in their local area to promote lasting social connections with other participants. These experiences help us to understand the lack of effect on social support and feelings of loneliness observed in the feasibility trial [29]. It is also important to acknowledge that the absence of social support from friends and family due to social distancing restrictions and resulting increases in loneliness and social isolation among new parents during the COVID-19 pandemic [15, 28] may have limited the impact that M4M-online was able to have on social outcomes. Challenges to social engagement have also been identified in online singing interventions with other populations with a key barrier being technological limitations to social interactions and connectedness [39, 43]. A study comparing experiences of moving from in-person to online choir sessions during the pandemic also identified limited opportunities for group and social bonding through the lack of real-time singing, harmonising and the act of singing together as a group within a shared physical space [45]. For mothers in our study and in studies with people experiencing chronic health problems [39, 43], however, the focus on singing whilst muted allowed some participants to express themselves more openly, particularly those who lacked confidence in their singing abilities. This highlights that different considerations might be needed for adapting and promoting engagement with online creative health interventions compared to creative activities for those who are already actively engaged in creative pursuits.

### Strengths and limitations

This study builds on and helps to interpret the findings from a feasibility trial on the impact of online singing groups for new mothers experiencing PND. It provides an in-depth understanding of how and why online singing groups helped reduce PND, stress and anxiety symptoms in new mothers during the COVID-19 pandemic as well as why social support and loneliness outcomes were not strongly impacted. It also formalises the identification and comparison of active ingredients of a creative health programme delivered across different formats with a theory-based approach.

The study is not without limitations. Whilst attempts were made to include all women who participated in the feasibility trial, 11 women did not respond to the invitation to take part. The current study sample, while being of a similar age to the overall trial sample, was less heterogeneous and contained fewer women from ethnic minority groups, those who were single, and those with fewer educational qualifications. Their experiences may have been less positive or different to the views and experiences expressed here. Finally, it was not possible to elucidate potential biological mechanisms through qualitative interviews, however previous research to support people affected by cancer identified increases in cytokines and reductions in cortisol and oxytocin levels following choir participation [46] and reductions in cortisol have been found in new mothers after participating in singing groups [20]. This suggests that singing can produce positive biological responses and warrants further investigation for women experiencing postnatal depression.

## Conclusions

We identified key programme features (‘active ingredients’) of an online group singing programme that helped to support new mothers experiencing postnatal depression. These features triggered a range of psychological, social and behavioural responses (‘mechanisms of action’) that led to improved mental health. We also compared online and in-person delivery formats to determine the likely “core ingredients” of the M4M programme. These findings can be used to (i) inform the design of future online creative health interventions to support mental health and maximise engagement and impact and (ii) tailor in-person activities for remote delivery to extend geographical reach and support populations who may struggle to attend in-person.

## Electronic supplementary material

Below is the link to the electronic supplementary material.

Supplementary Material 1

Supplementary Material 2

## Data Availability

The datasets used during the current study are available from the corresponding author upon reasonable request.

## Abbreviations

M4M: Breathe Melodies for Mums
COVID-19: Coronavirus disease 2019
EPDS: Edinburgh Postnatal Depression Scale
INNATE: INgredients iN ArTs in hEalth Framework
KCL: Kings College London
NCT: National Childbirth Trust
PND: Postnatal depression
SHAPER: Scaling-up Health-Arts Programme
UCL: University College London
