# Supplementary material for: A qualitative exploration of active ingredients and mechanisms of action of an online singing programme with mothers experiencing postnatal depression during the COVID-19 pandemic: SHAPER-PNDO study
Source: BMC Psychol. 2024 Nov 30;12:714. doi: 10.1186/s40359-024-02213-7 (PMC11608468; doi:10.1186/s40359-024-02213-7)
Supplement: Supplementary file 1 — Supplementary Material 1 [file 40359_2024_2213_MOESM1_ESM.docx]

**Topic guide - M4M-online participants**

| **Introduction** | Explaining purpose and context of the interview  Reminding about the research ethics (e.g. that information is reported anonymously, that audio recordings will be destroyed after analysis of anonymised transcripts) |
| --- | --- |
| **Main body** | ***Lived experiences of postnatal depression (PND)***  We’d like to talk a bit about your experiences so far as a mum – could you tell us how it’s been for you? What have been the highlights? The challenges? What’s it felt like?  As you know, *Melodies* is for mums experiencing symptoms of postnatal depression such as low mood, anxiety or stress. Could you share your experiences of these symptoms?  ***Singing and PND***  So thinking about the online singing sessions –  Could you tell me why you decided to take part in *Melodies for Mums*?  Could you tell us about your experiences at *Melodies* over the past 6 weeks?  How has the programme impacted, if at all, your *feelings*? Could you give us an example? What was it about the programme that made that impact?  How has the programme impacted, if at all, how you *think of yourself* as a mum? Could you give us an example? What was it about the programme that made that impact?  How has the programme impacted, if at all, how you *behave* as a mum? Could you give us an example? What was it about the programme that made that impact?  How would you describe *Melodies* to another mum experiencing low mood, anxiety or stress? [prompt: would you recommend to others]  What has it meant to you to take part in *Melodies*? [prompt: How has it supported you, if at all? How has it met the challenges, if at all, of motherhood and PND?]  If you could be involved in the design of *Melodies*, is there anything you might want to change about it, if anything at all?  ***Transition to close***  Is there anything else that you’d like to mention? |
| **Close** | Thanks; remind how data will be used; contact information for the research team; contact information for further support |
