# Supplementary material for: A qualitative exploration of active ingredients and mechanisms of action of an online singing programme with mothers experiencing postnatal depression during the COVID-19 pandemic: SHAPER-PNDO study
Source: BMC Psychol. 2024 Nov 30;12:714. doi: 10.1186/s40359-024-02213-7 (PMC11608468; doi:10.1186/s40359-024-02213-7)
Supplement: Supplementary file 2 — Supplementary Material 2 [file 40359_2024_2213_MOESM2_ESM.docx]

#

# Active ingredients worksheet comparing the Melodies for Mums online and in-person programmes

The following worksheet compares the active ingredients of two interventions: The Breathe Melodies for Mums (M4M) online and M4M in-person programme for women experiencing postnatal. The ingredients of the online-programme are listed in the left-hand column and the ingredients of the in-person programme are listed in the right-hand column.

**PART ONE: PROJECT**

|  | M4M-online | M4M in-person |
| --- | --- | --- |
| ATTRIBUTES | | |
| 1. What is the format of your activity? | | |
| Mode  Describe the form in which the activity occurs | *Virtual* | *In-person* |
| Synchroneity  Explain the degree to which the activity or engagement occurs in real-time for participants | *Live/real-time singing combined with pre-recorded tracks and technical set-up with microphone for artist* | *Live/real-time* |
| Activity level  Tell us about the extent to which the activity requires active participation | *Active/participatory* | *Active/participatory* |
| Additional comments | *Participants were also invited to connect socially via a WhatsApp group* |  |
| 1. How much (i.e., what ‘dose’) of your activity is received by participants? | | |
| Frequency  Explain how often the activity occurs | *Once a week* | *Once a week* |
| Duration  Describe the length of one dose/exposure of the activity in time | *1hr* | *1hr* |
| Maintenance  Tell us over what time periods the activity continues | *6-weeks* | *10-weeks* |
| Additional comments |  |  |
| 1. What is the design (structural plan) of your activity? (This may or may not be adaptable.) | | |
| Structure  Describe how the activity is organised in its delivery | *Semi-structured: unstructured online socialising/welcome, warm-up which consisted of stretching, breathing and throat exercises, then into structured singing and time for unstructured socialising again at the end* | *Semi-structured: unstructured online socialising/welcome, warm-up which consisted of stretching, breathing and throat exercises, then into structured singing and time for unstructured socialising again at the end* |
| Guiding  Explain whether the activity is taken in a particular direction to reach a goal or outcome | *Taught. Lyrics were shared in the online meeting chat for participants to follow* | *Taught* |
| Project approaches  Describe approaches or techniques employed within the activity that characterise it | *Singing predominantly on mute with the singing lead unmuted. Accompanying backing tracks* | *Participants copied the singing lead to learn the songs and then sang together, in harmony and in rounds* |
| Personalisation  Explain whether the activity is designed to meet participants' needs or preferences | *A range of songs were used including upbeat, emotional, fun and calming songs to support the mental health of participants and to give participants a range of songs to use in different situations with their babies. Songs are used from different cultures in different languages for inclusivity* | *A range of songs were used including upbeat, emotional, fun and calming songs to support the mental health of participants and to give participants a range of songs to use in different situations with their babies. Songs are used from different cultures in different languages for inclusivity* |
| Challenge  Describe the intended level of difficulty of the activity, including whether the difficulty can be adapted across participants | *Easy and accessible. Some of the songs were catchy, memorable and include repeated phrases or melodies. Use of backing tracks allowed participants to try harmonies and rounds* | *Easy and accessible. Some of the songs were catchy, memorable and include repeated phrases or melodies. Participants sang in harmonies and rounds.* |
| Goal orientation  Tell us about whether the activity is directed towards a particular aim or ends, including who sets goals | *Non goal focused* | *Non goal focused* |
| Feedback  Describe if and how evaluative information or reflections are included in the design of the activity | *Informal non-judgemental and positive feedback was provided to the group by the singing lead during the sessions. When participants were asked to come off mute the singing lead would ask how they were doing and invite them to say how they found it. The singing lead also commented on what they saw on the screen* | *Informal non-judgemental and positive feedback was provided to the group by the singing lead during the sessions* |
| Additional comments | *NB. Participants were on mute when they responded to the live online sessions and for the most part during singing could only hear the singing leader and backing tracks and not other participants. Participants came off mute occasionally to sing together and to chat* |  |
| 1. What is the artistic content of your activity?   (Please note that artistic resources are included in the next question) | | |
| Genre  Describe the primary branch of creative activity in which the activity(ies) is(are) categorised as well as any sub-categories | *Music and singing (familiar popular and songs from different cultures in different languages)* | *Music and singing (familiar popular and songs from different cultures in different languages)* |
| Multi-modality  Tell us if the activity also draws upon a range of different art forms or disciplines in its delivery | *N/A* | *N/A* |
| Activity type  The kind(s) of activity(ies) included that define(s) the creative engagement | *Group singing* | *Group singing* |
| Themes  Tell us if there is engagement with specific themes or subjects as part of the artistic content | *Singing leads could set their own repertoire which usually included a range of songs including positive and upbeat, inspiring, calming and emotional as well as songs from popular culture and songs from different cultures in different languages. Topics included songs about motherhood, and song lyrics were sometimes adapted to reflect experiences of motherhood. Songs were aimed predominantly at mothers rather than the babies but some children’s songs and lullabies were included* | *Singing leads could set their own repertoire which usually included a range of songs including positive and upbeat, inspiring, calming and emotional as well as songs from popular culture and songs from different cultures in different languages. Topics included songs about motherhood, and song lyrics were sometimes adapted to reflect experiences of motherhood. Songs were aimed predominantly at mothers rather than the babies but some children’s songs and lullabies were included* |
| Additional comments |  |  |
| 1. What resources (physical, conceptual or informational) materials are used/employed in the delivery of your activity? | | |
| Activity consumables  List activity resources that can be used up or depleted | *None* | *None* |
| Props  List items such as objects or furnishings employed within the activity | *Participants were welcome and encouraged to bring their own props to the online singing sessions including homemade instruments such as shakers, muslin clothes and scarfs. Singing leads had microphones and headphones connected to zoom. Some of them had mixing desks and loop pedals. The singing lead sometimes played an instrument (e.g. ukelele)* | *Children’s shakers and toys, cushions, blankets, chairs and mats. The singing lead sometimes played an instrument (e.g. ukulele)* |
| Products  List tangible outputs of the activity | *Participants had access to the password-protected Breathe Melodies for Mums participant hub, hosted via the Breathe Arts Health Research website. The hub included a library of song recordings used in sessions to listen to at home and to share with the wider family, lyrics and signposting links to additional mental health support provided by charitable organisations and the NHS* | *Recorded songs on Soundcloud were available after the singing sessions* |
| Performances  List intangible outputs of the activity (incapable of being perceived by the sense of touch) | *N/A* | *The mothers recorded some simple songs that they sang together* |
| Additional comments |  |  |
| 1. Are there any activities that are integrated into the arts/cultural activity?   (NB. Collaborations or co-produced projects which form part of the project’s set-up shall be returned to in part three.) | | |
| Psychosocial support  Describe any integrated professional techniques and/or resources designed to support mental health, wellbeing, experiences of psychological disorders and/or quality of life | *Stretching and breathing techniques were often used in warm-ups. Some groups also used Makaton* | *Stretching and breathing techniques were often used in warm-ups* |
| Allied therapies  Describe any integrated therapeutic techniques that treat or manage physical disability, malfunction, pain or stress and tension via physical methods | *N/A* | *N/A* |
| Health education  Detail any integrated learning experiences designed to help individuals and communities improve their health by increasing their knowledge or influencing their attitudes | *The Breathe Melodies for Mums participant hub included signposting links to additional mental health support provided by charitable organisations and the NHS. The Breathe staff member supporting the session provided 1:1 bespoke and geographically targeted signposting to services for participants if identified as requiring additional support or on request* | *The sessions did not include any explicit health education, but the location of a Children’s Centre contained posters and leaflets about maternal and infant health* |
| Spiritual or holistic practice  Detail any integrated experiences relating to religious, spiritual or mind-body practices | *N/A* | *N/A* |
| Socially-engaged practice  Describe any integrated programmes designed to engage with social issues or that seek social or political change | *N/A* | *N/A* |
| Additional comments |  |  |
| ENGAGEMENT | | |
| 1. Do you employ/use/engage with any objects, actions, materials or experiences that activate the senses as part of the activity (sensory stimuli)? | | |
| Sight (Vision)  Explain if participants perceive objects or imagery by use of their eyes as part of the activity | *Song lyrics were posted in the chat function. Participants are encouraged to keep their videos on to increase engagement between them* | *N/A* |
| Hearing (Auditory)  Explain if participants perceive stimuli by ear as part of the activity | *Music/backing tracks to sing along with. Participants listened to the singing lead and sometimes sang simple songs off mute together* | *Participants listened to the singing lead and to each other singing* |
| Smell (Olfactory)  Explain if participants perceive odour or scent through the nose as part of the activity | *N/A* | *N/A* |
| Taste (Gustatory)  Explain if the act of tasting food or drink is part of the activity | *N/A* | *N/A* |
| Touch (Tactile)  Explain if the body (i.e., hand, finger) is put in contact with something (i.e., an object or person) as part of the activity | *Participants may have had to hold their laptops or mobile phones. Participants held and interacted with their babies* | *Participants held and interacted with their babies. Cushions and blankets to sit on and shakers were available* |
| Additional comments |  |  |
| 1. Do you employ/use/engage with any objects, actions, materials or experiences that activate cognitive and/or creative processes as part of the activity (cognitive stimuli)? | | |
| Involvement of the imagination  Explain if mental images or concepts beyond the senses are part of the activity | *Participants were sometimes encouraged to suggest or share songs from their own cultural background or that they had grown up with* | *Participants were encouraged to engage with the images of the songs and contribute suggestions to group compositions* |
| Emotional stimuli  Explain if there is something about the activity or aspects of it that brings about affective states of consciousness (feelings) | *A range of songs to stimulate different emotions. Positive music used to uplift participants, songs about motherhood which could prompt an emotional response, calm and gentle songs to relax participants and humorous songs to make participants laugh* | *A range of songs to stimulate different emotions. Positive music used to uplift participants, songs about motherhood which could prompt an emotional response, calm and gentle songs to relax participants and humorous songs to make participants laugh* |
| Cognitive stimulation  Explain if there is something about the activity or aspects of it that prompts mental processes of perception, memory, judgment, and reasoning (contrasted with emotional processes) | *Participants learned lyrics of new songs and songs in different languages* | *Participants learned lyrics of new songs and songs in different languages* |
| Aesthetic engagement  Describe if participants engage with the activity through subjectively sensing something as beautiful | *Participants were encouraged to see the songs they sang as beautiful* | *Participants were encouraged to see the songs they sang and created as beautiful* |
| Pleasure  Explain if there is something about the activity or aspects of it that brings about feelings of pleasure | *There were often moments of fun, joy and laughter during the singing sessions prompted by specific songs, informal chat and the singing lead entertaining the group* | *There were often moments of fun, joy and laughter during the singing sessions prompted by specific songs and informal chat and the singing lead entertaining the group* |
| Participant choice  Describe if participants make selections from a number of possibilities based on preference or under guidance as part of the activity | *Participants were given some choice in the sessions, such as between two different songs to sing, and they were encouraged to bring songs from their own cultural background to the sessions* | *Participants were given some choice in the sessions, such as between two different songs to sing, and they were encouraged to bring songs from their own cultural background to the sessions* |
| Anything else? |  |  |
| 9. Are any physical bodily motions or actions employed as part of the activity? | | |
| Proprioception (or kinaesthesia)  Explain if heightened awareness of the body's position and movements is part of the activity, and whether it’s free or guided | *Participants became aware of their bodies through breathing, and stretching activities during the warm-up exercises* | *Participants became aware of their bodies through breathing, and stretching activities during the warm-up exercises* |
| Movement  Tell us if bodily movement is prompted by the activity | *Stretching activities were used in the warm up at the start of the session* | *Stretching activities were used in the warm up at the start of the session* |
| Physical exercises  Explain if any exercises are employed as part of the activity to promote bodily fitness and strength | *N/A* | *N/A* |
| Additional comments |  |  |

**PART TWO: PEOPLE**

| SOCIAL COMPOSITION | | |
| --- | --- | --- |
| 1. Who are the people involved in the activity (social diversity)? | | |
| Presence of others  Detail the number of people present and/or the size of the group that the activity entails | *A group of between 3-15 people* | *A group of between 3-12 people* |
| Shared attributes  Describe whether individuals engaging together in the activity have characteristics in common | *All were mothers aged 18+ scoring 10 or more on the Edinburgh postnatal depression scale (EPDS) with babies aged between 0-9 months at the start of the programme* | *All were mothers aged 18+ scoring 10 or more on the Edinburgh postnatal depression scale (EPDS) with babies aged between 0-9 months at the start of the programme* |
| Distinct attributes  Describe the diversity of individuals engaging together in the activity | *There was some racial, geographical and socioeconomic diversity within the groups* | *There was some racial and socioeconomic diversity within the groups* |
| Personal attributes  Describe any other personal qualities that participants have which inform how they engage with the activity | *N/A* | *N/A* |
| Additional comments |  |  |
| 1. Do participants have any previous experiences which are relevant to how they engage with the activity? | | |
| Activity experience  State whether individuals engaging have previous experience(s) of the activity | *Several participants had previous experience of singing in choirs, while others had no formal group singing experience* | *Several participants had previous experience of singing in choirs, while others had no formal group singing experience* |
| Health experience  Outline whether individuals engaging have previous experience or knowledge of specific health conditions, healthcare in general, or health outcomes | *No previous experience of arts activities in healthcare contexts was required. An official diagnosis of postnatal depression was not required to participate in the programme* | *No previous experience of arts activities in healthcare contexts was required. An official diagnosis of postnatal depression was not required to participate in the programme* |
| Lived experience  Tell us whether individuals engaging have personal experiences relevant to who the activity is tailored for | *All participants had some experience of low mood, stress or anxiety symptoms and had recently accessed maternity healthcare during the COVID-19 pandemic* | *All participants had some experience of low mood, stress or anxiety symptoms and had recently accessed maternity healthcare* |
| Relationship to others  Explain the kind of relationships the individuals engaging together in the activity have to one another | *Mothers joined sessions with their babies from home* | *Mothers brought their babies along to the group sessions* |
| Additional comments | *N/A* | *N/A* |
| 1. Are there any social interactions (face to face or digital) part of or integrated into the activity? | | |
| Shared focus  Explain if and how attention is given collectively to an object, activity, thought or person/people as part of the activity | *Joint focus on the singing leader during the sessions. Mothers could also see themselves on the screen with their babies and see other mums with their babies – participants were encouraged to keep cameras on* | *Joint focus on the singing leader during the sessions* |
| Shared activity  Explain if and how participants cooperate or collaborate as part of the activity | *The full group sang together with the singing leader, predominantly on mute so they could only hear the singing lead. Some singing was done off mute together. Songs sometimes required rounds or harmonies, call and response* | *The full group sang together with the singing leader. Songs sometimes required rounds or harmonies, call and response* |
| Social exchanges  Describe the social elements of the activity itself that involve interaction with others | *Participants were given time to chat at the beginning and end of the session with one another and the singing lead. When participants talked to one another they could share their problems and experiences and participants and the singing lead observed and commented on positive behaviours in each other’s babies (e.g. babies laughing or smiling) on the screen* | *Participants were given time to chat at the beginning and end of the session with one another and the singing lead* |
| Structured social time during activity  Describe any aspects of the activity that encourage the formation of social relationships or socialising | *Facilitated introductions and welcome at the first session and time to chat at the beginning and end of each session. The Breathe staff member set up a WhatsApp group for participants to form social relationships during the programme* | *Facilitated introductions and welcome at the first session and time to chat at the beginning and end of each session.* |
| Structured social time outside of activity  Detail any planned time outside of the core activity delivery used to encourage the formation of social relationships or socialising | *N/A* | *At the end of the 10 weeks, a WhatsApp group was created between the mothers who communicated between the sessions* |
| Communications  Tell us if and how those in leadership/management communicate with participants in the lead up to and after the activity | *Participants were sent an email or text with a welcome pack and the joining link to the Zoom session by the supporting Breathe staff member. ‘Check in’ emails and calls were also made to participants to see how they were doing. Participants received weekly text reminders to attend sessions. Access to the Breathe Melodies for Mums participant hub was shared by the supporting Breathe staff member* | *Participants were sent information from the administrators reminding them about sessions, but did not have direct contact from the singing lead* |
| Additional comments |  |  |
| 1. Are there social exchanges (face to face or digital) that that are not planned as part of the activity | | |
| Unstructured social time during activity  Describe if space is provided during an activity for participants to informally socialise | *Participants were invited to join the Zoom room prior to the session starting if they wanted more time to chat and connect informally* | *At the beginning and end of sessions participants had a few minutes to say hello and chat to the others in the class* |
| Unstructured social time outside of activity  Detail if social time or social activities are shared between participants outside of the activity, without formal guidance | *Participants could communicate with one another via a WhatsApp group if they had opted in. If local to one another some participants also met up* | *Participants would often go for a coffee after the sessions or proactively arranged to meet outside of the sessions* |
| Additional comments |  |  |
| ACTIVITY FACILTATION | | |
| 14. What kind of facilitation (i.e., the people who lead, guide or facilitate the participant-facing aspects of the activity and not the administrative aspects) is employed? | | |
| Facilitator(s)  Explain who facilitates the activity during its delivery and what form this facilitation takes | *A singing lead facilitated the sessions, and a Breathe co-facilitator supported the technology and practical/social support for participants* | *A singing lead facilitated the sessions, and a co-facilitator provided practical/social support to participants and their babies* |
| Co-production  Describe if the activity involves actively including participants in the process of delivering and facilitating it | *Participants were invited to bring some song suggestions to the classes* | *Participants were invited to bring some song suggestions to the classes and help in the creation of group songs* |
| Number  Detail the number of people who facilitate or lead the activity | *One singing lead and one facilitator* | *One singing lead and one facilitator* |
| Professionalisation  Tell us about whether the person/people who facilitate the activity identify as professional(s) within their specific field/domain | *Professional singers who may have performed professionally and ran other singing activities, especially in arts and health* | *Professional singers who also performed professionally and ran other singing activities* |
| Training  Explain if the person/people who facilitate the activity have professional training (i.e., domain-specific skills) | *Singing leads were supported with regular briefing, debriefing and training opportunities delivered by Breathe focussed on their delivery/practice but within the context of working with participants experiencing poor perinatal mental health. Supporting Breathe staff were experienced in working with vulnerable people and were trained in safeguarding* | *The facilitators had professional training as workshop leaders and professional musicians received a series of training sessions with the research leads, a perinatal psychiatrist and a health visitor preparing them for working with mothers with PND* |
| Consistency  Explain whether facilitation changes or stays the same | *There were a number of singing leads and facilitators but one singing lead and one Breathe staff member is assigned to work with the same group for the entire 6-weeks. Replacements would happen if someone was away or unwell* | *There were a number of singing leads and facilitators but one singing lead and one facilitator is assigned to work with the same group for the entire 10-weeks. Replacements would happen if someone was away or unwell* |
| Additional comments | *Both singing leads and Breathe staff were supported by an Employee Assistance Programme providing confidential, generalised support and advice on all areas of their lives* |  |
| 15. If there is a facilitator, what experience do they bring to the delivery of the project? | | |
| Activity experience  Tell us about the amount of previous domain-specific experience, knowledge or skills the facilitator(s) have | *The singing leads had past experience in leading similar workshops for mums and babies and/or extensive community music leadership* | *The singing leads had past experience in leading similar workshops for mums and babies as well as extensive community music leadership* |
| Health experience  Outline the amount of previous experience or knowledge the facilitator(s) have in relation to specific health conditions, healthcare in general, or health outcomes | *The singing leads were specialist arts and health leads, although not necessarily working with PND before the project* | *The singing leads did not have specific knowledge of PND before the project or any declared lived experience* |
| Lived experience  Tell us if the facilitator(s) have personal experiences relevant to who the activity is tailored for | *Some of the singing leads had experience of being a mother* | *Some of the singing leads had experience of being a mother* |
| Relationship to others  Tell us if the facilitator(s) have any pre-existing relational experiences of engaging with participants | *The singing leads didn’t know anyone in the group before the sessions began* | *The singing leads didn’t know anyone in the group before the sessions began* |
| Additional comments |  |  |
| 16. In what style/manner is the activity is delivered and what artistic practice is drawn upon? | | |
| Technique  Explain whether the activity facilitator(s) draw on approaches or technical skills that are characteristic of one’s domain-specific field | *Vocal pedagogy. Tracks were pre-recorded to enable the recreation of harmonies and rounds by participants online. This required a strong technical set up* | *Vocal pedagogy (singing lead)* |
| Personal attributes  Detail any additional qualities that the facilitator(s) bring to the activity that informs how it is delivered | *The singing leads had excellent communication skills and were good listeners. They were enthusiastic and fun. The supporting Breathe staff member was empathetic and intuitive to participants requiring additional support to engage.* | *The singing leads had excellent communication skills and are good listeners. They were enthusiastic and fun* |
| Values-directed focus  Explain if and how the facilitator(s) deliver the activity based on specific ethical values | *The singing leads sought to be inclusive by fostering a space where all were able to ask questions, where there was no ‘right’ way to sing. They respected participants and sought to be responsive to participant needs. Mothers were encouraged to accept all potential disruptions from their babies without judgement and to participate or watch as required to fit in with feeding or changing or comforting of their infants.*  *Participants were encouraged to have their cameras on but informed that it was okay to have them off momentarily for moments like feeding and changing* | *The singing leads sought to be inclusive by fostering a space where all were able to ask questions, where there was no ‘right’ way to sing or to engage. They respected participants and sought to be responsive to participant needs. Mothers were encouraged to accept all potential disruptions from their babies without judgement and to participate or watch as required to fit in with feeding or changing or comforting of their infants* |
| Outcomes-directed focus  Explain if and how the facilitator(s) focus on health, educational, or aesthetic goals as part of the activity | *The focus was on learning songs to support mental health, improve confidence interacting with their babies and bonding with their babies* | *The focus was on learning songs to support mental health, improve confidence interacting with their babies and bonding with their babies* |
| Person-centred focus  Explain if and how the facilitator(s) consider participant preferences, needs, and values to deliver the activity | *The singing lead and Breathe staff member were friendly, warm and welcoming and made an effort to speak to every individual participant, so they felt welcomed. Participants were asked if they had any particular needs during and in between the sessions. If participants were struggling to engage for any reason such as technical issues, the Breathe staff member supported them via direct message or phone call* | *The singing lead and facilitator were friendly, warm and welcoming and made an effort to speak to every individual participant, so they felt welcomed. Participants were asked if they had any particular needs during and in between the sessions. Facilitators took the baby or watched the baby if the participant needed a break* |
| Autonomy-directed focus  Explain if and how the facilitator(s) provide participants with autonomy as part of the activity | *The singing lead often asked participants whether they would like to request songs* | *Participants were given some autonomy in how they engaged (e.g. which instruments to use), how to move their babies, and some selection over songs* |
| Equality, Diversity and Inclusion  Explain if and how the facilitator(s) consider fair treatment and equal opportunities to deliver the activity | *The singing lead actively chose songs in different languages and from different cultures, sharing origin, meaning and context, and encouraged mothers to bring their own songs to the classes too. Song lyrics were considered carefully for inclusivity. On registration, participants were asked whether they had any access needs. An access budget was included in the programme budget should participants require support to join such as via mobile data* | *The singing lead actively chose songs in different languages and from different cultures, sharing origin, meaning and context, and encouraged mothers to bring their own songs to the classes too* |
| Safety  Explain if and how the facilitator(s) consider the safety of participants in how the activity is delivered | *The supporting Breathe staff member was trained in safeguarding and able to respond to any concerns for participants in the group. Participants were encouraged to keep cameras on for this reason. The singing lead and Breathe staff member wore Breathe t-shirts so they were clearly identifiable as leading the session on the screen.* | *The singing lead reported back to the research team if there were any concerns for the mothers in the group or their babies that they picked up on during the classes* |
| Tailoring  Explain if and how the facilitator(s) personalise or adapt the activity to meet the needs of participants | *The singing activities were tailored to the group dynamics such as lively if participants seemed energised, or relaxing if participants were feeding their babies or their babies were sleeping. The backing tracks provided different ways for mothers to engage depending on their level of confidence e.g. participating in rounds or adding harmonies* | *The singing lead provided different ways for mothers to engage depending on their level of confidence e.g. adding harmonies or simple instruments, participating in rounds* |
| Additional comments |  |  |
| 17. Are there additional staff or other people that support, co-lead or are present at the activity? | | |
| Presence of volunteers  List any unpaid staff who support or co-lead the delivery of the activity and explain what they do to support | *N/A* | *N/A* |
| Presence of healthcare professionals  List any healthcare professionals and how they support or co-lead the delivery of the activity | *Healthcare professionals were not present in the workshops, but there was a safeguarding protocol in place for any concerns to be addressed and escalated if required* | *Healthcare professionals were not present in the workshops, but there was a safeguarding protocol for any concerns to be reported back to mothers’ GPs.* |
| Presence of others  Detail whether there are any other staff or people and how they support, co-lead or are present for the delivery of the activity | *A Breathe staff member always attended the groups to provide technical and social support and troubleshoot any problems. This allowed the singing lead to focus on creative delivery* | *A facilitator always attended the groups to provide practical and social support and support with the babies. This allowed the singing lead to focus on creative delivery* |
| Additional comments |  |  |

**PART THREE: CONTEXTS**

| SETTING | | |
| --- | --- | --- |
| 1. What circumstances, objects, and conditions make up the surrounding environment of the activity? | | |
| Location  Describe the place where the activity is delivered | *Online using Zoom – participants were in their own homes* | *Indoors at a Children’s community centre* |
| Basic features  Describe the functional aspects of where the activity takes place and how the room(s)/space(s) are arranged | *Participants engaged via Zoom from their laptops or mobile phones within their own homes where they all had different basic features however often participants were able to see themselves and their babies on screen, the singing lead and other participants and their babies.* | *The venues were warm and well lit. There were good basic facilities. Soft blankets, cushions and mats were placed on the floor for mothers and babies to sit on. Chairs were also available for participants to sit on. Toys and shakers were available for the babies to play with. Participants tended to form a semi-circle with the singing lead at the front* |
| Attractiveness  Explain whether the environment of the activity is perceived as beautiful, attractive or pleasing to the eye. Note any modifications made. | *The singing lead and Breathe member of staff wore Breathe branded t-shirts* | *The environment was colourful, comfortable and informal with the focus on children and babies* |
| Situation  Outline the geographic and/or socioeconomic features of where the activity takes place | *The singing group took place online in participant homes. Participants were spread geographically across the UK* | *The singing groups took place in South London. Some of the children’s centres were in deprived areas* |
| Time and day  Detail when the activity takes place and if this changes | *Singing groups took place at different times of the day and different days of the week for different groups, however the day and time was consistent for each group. The groups did however always take place between 10am and 4pm and on a weekday (not weekend)* | *Singing groups took place at different times of the day and different days of the week for different groups, however the day and time was consistent for each group. The groups did however always take place between 10am and 4pm and on a weekday (not weekend)* |
| Access  Explain the methods used and the means or opportunities available to find and participate in an activity | *All participants accessed the singing online via their mobile phones or laptops. An access budget was available should participants require support to join such as via mobile data.* | *Most participants travelled between 5 to 90 minutes by either walking, driving or using public transport to get to the groups.* |
| Privacy  Tell us if the location of the activity is accessible by anyone (including those not engaging) or if it is only open to those who are part of the activity | *Online Zoom meeting – only participants with a link could join and the meeting room was closed so the Breathe staff member was in control of who joined.* | *Private room in a children’s community centre* |
| Additional comments |  |  |
| 19. What is the atmosphere (character, feeling, or mood) like where the activity takes place? | | |
| Comfort  Explain the degree to which the setting of the activity elicits a sense of ease, safety and relaxation | *Singing on mute was comforting for some participants who felt more relaxed. Singing at home was easy and comfortable for most participants.* | *The play mats, cushions and toys supported a comforting mood. The children’s centre location helped participant’s feel safe and relaxed.* |
| Belonging  Explain the degree to which the setting and environment elicits a feeling of being included | *Being able to see other mums and babies on screen helped foster a sense of inclusion, however there was the option to turn off camaras if necessary. Singing together off mute aimed to foster a sense of collaboration however many participants reported that this did not work well due to noise interference.*  *There was an optional WhatsApp group to encourage socialising outside of the groups and participants were given password protected access to the Breathe Melodies for Mums participant hub* | *Every effort was made to ensure all felt welcome and comfortable to participate. Participants were encouraged to all sit /stand together on the play mats and chairs in the centre of the room* |
| Familiarity  Explain the degree to which the setting of the activity is known or unknown by participants | *Most participants had previous experience of using Zoom or other online activities. Participants engaged from their own homes in familiar surroundings* | *Many people were fairly local to the Children’s community centre. Some people did travel further and it was outside of their local community* |
| Ambiance  Describe the mood or tone of the surroundings where the activity takes place | *The atmosphere was positive, welcoming, calm but also fun. Participants engaged from their own homes in familiar surroundings* | *The atmosphere was positive, welcoming, calm but also fun* |
| Organisation  Explain how the delivery and management of the activity is perceived by participants | *Well organised with text reminders and email joining links sent prior to each session and a Breathe staff member was on hand during the session to post lyrics into the chat and support with technology issues* | *Well organised with reminder texts and emails about classes each week and clear signposting and welcoming at the venue* |
| Additional comments |  |  |
| PROJECT SET-UP | | |
| 1. What economic resources (if any) are connected to the activity and its delivery? | | |
| Participant charges  Describe any monetary transactions connected to or part of the activity, and if there are any support structures in place to ensure equal distribution of monetary resources | *It was free for participants to attend* | *It was free for participants to attend* |
| Project funding  Explain if and how the activity is delivered using monetary resources obtained through external organisations, individuals, trusts, charities or the government to be used for the purpose of delivering the activity | *Funded for the purposes of the research study by a research grant* | *Funded for the purposes of the research study by a research grant* |
| Fees  Describe if any people involved in the delivery of the activity are paid for their time | *All delivery staff were paid for their time* | *All delivery staff were paid for their time* |
| Longevity  Describe the duration of the activity across time and whether the activity can be upheld and supported with the economic resources available | *The intervention was run for the research study. It has since been taken on in a similar form through healthcare and grant funding* | *The intervention was run for the research study.* |
| Environmental sustainability  Tell us if best use of resources are made in view of what may be harmful to the environment | *Lyrics were posted in the chat function rather than printed and posted. There were no other disposable resources* | *There were no lyric sheets or other disposable resources* |
| Additional comments |  |  |
| 21. What person, people, group(s) and/or company(ies) is/are in charge of organising the management of the activity? | | |
| People  Describe if there are any people ‘behind the scenes’ of the participant-facing aspects of the activity | *The classes were supported behind the scenes by research assistants and researchers as well as the singing leads and Breathe staff members involved with running the classes* | *The classes were supported behind the scenes by research assistants and researchers as well as the musicians involved with running the classes* |
| Affiliation  Explain if and how the activity is connected to an organisation or institution | *Led by Breathe Arts Health Research* | *Led by the Royal College of Music* |
| Branding  Describe the language, visual imagery, ethos and/or symbols that represent any connected organisations, institutions and/or the activities | *The logo for the organisation delivering the singing sessions combined an artistic and scientific aesthetic depicting a doodled brain using different shades of green. The language was welcoming, focussed on symptoms rather than diagnosis, and promotes emotional and mental wellbeing whilst also linking the impact of the activities to evidence and research. The singing lead and Breathe staff member wore Breathe t-shirts* | *The logo for the organisation delivering was the RCM logo in black and red* |
| Collaboration  Describe any partnerships with other organisations involved in the delivery of the activity | *N/A* | *N/A* |
| Patient and Public Involvement  Describe if the activity involves actively including participants or the public in the process of designing and organising the activity | *Building on key ingredients from the co-designed Breathe Melodies for Mums in-person delivery model, the online programme was developed using feedback from stakeholders who had previously taken part in other Breathe online arts interventions* | *The programme was developed and refined together with artists, researchers, and women experiencing postnatal depression* |
| Additional comments |  |  |
| 22. How do participants find out about or become enrolled into the activity? | | |
| Formal referral  Explain if there is a referral into the activity from professional services such as via established organisations or schemes | *Self-referral however healthcare professionals could signpost people to the group* | *Self-referral however healthcare professionals could signpost people to the group* |
| Informal referral  Explain if there is a referral into the activity from personal, social group, or community connections or networks | *Friends and relatives could signpost women to the groups. Information was shared via social media* | *Friends and relatives could signpost women to the groups. Information was shared via social media* |
| Choice  Tell us who decides if the participant will enrol in the activity | *Participant choice based on meeting eligibility criteria* | *Participant choice based on meeting eligibility criteria* |
| Advertising  Describe how participants find out about the activity via publicity materials, and whether these materials are targeted for particular groups | *Fliers and posters advertised within GP practices, baby weigh clinics, children’s centres, libraries and shops, advertising via social media, word of mouth, and sending out information to healthcare professionals for signposting* | *Fliers and posters advertised within GP practices, baby weigh clinics, children’s centres, libraries and shops, advertising via social media, word of mouth, and sending out information to healthcare professionals for signposting* |
| Additional comments |  |  |
| 23. Do you signpost or refer to any services, resources, support, or advice beyond the activity itself? | | |
| Inter-sector signposting  Describe any resources and information provided about other arts or cultural activities that may be suitable for the participant(s), or if participants are directly recruited into such activities | *Not formally, but participants often shared their experiences of other arts/cultural activities with others in the group. As participants were joining from various geographies, Breathe staff tended to signpost participants to other classes and arts/cultural activities on response tailored to their location* | *At the end of the course, participants were given a leaflet about other classes and arts/cultural activities in the area they could consider joining* |
| Health-sector signposting  Describe any resources and information provided about healthcare or support for mental or physical health, or if participants are directly recruited into such activities | *The Breathe Melodies for Mums participant hub included signposting links to additional mental health support provided by charitable organisations and the NHS, and a signposting sheet with links to these services was provided at the end of the programme. The Breathe staff member supporting the session could provide 1:1 bespoke and geographically targeted signposting to services for participants if identified as requiring additional support or on request.* | *Mothers were provided with fliers about PND and support available to them, and the venue the classes took place in had further resources and leaflets on maternal and infant health* |
| Social signposting  Describe any resources and information provided about social support services/activities, or if participants are directly recruited into such activities | *N/A* | *N/A* |
| Other-sector signposting  Describe any resources and information provided about other sectors, or direct recruitment into such activities | *N/A* | *N/A* |
| Safeguarding referral  Describe if and how action will be taken to protect participants from emotional or physical harm if it is needed | *If there was any concern over the welfare of mothers or their babies, either through observation at the classes or results on the questionnaires they completed as part of the research, they were contacted by the research team to discuss their wellbeing and safeguarding protocols followed if necessary.* | *If there was any concern over the welfare of mothers or their babies, either through observation at the classes or results on the questionnaires they completed as part of the research, they were contacted by the research team to discuss their wellbeing, and (if it was felt to be necessary), their GP was also contacted.* |
| Additional comments |  |  |

**FURTHER COMMENTS**

| Use the space below to note any further comments in relation to identifying the active ingredients of your arts or cultural activity. |
| --- |
|  |

5.2 Colour chart comparing ingredients of M4M-online vs M4M in-person

Key

|  | Similar active ingredients |
| --- | --- |
|  | Some differences, some similarities |
|  | Different active ingredients |
|  | N/A for activity |

| PROJECT | |
| --- | --- |
| Attributes | |
| Format | **Mode** |
|  | **Synchroneity** |
|  | **Activity level** |
| Dose | **Frequency** |
|  | **Duration** |
|  | **Maintenance** |
| Design | **Structure** |
|  | **Guiding** |
|  | **Project approaches** |
|  | **Personalisation** |
|  | **Challenge** |
|  | **Goal orientation** |
|  | **Feedback** |
| Artistic content | **Genre** |
|  | **Multi-modality** |
|  | **Activity type** |
|  | **Themes** |
| Activity resources | **Activity consumables** |
|  | **Props** |
|  | **Products** |
|  | **Performances** |
| Integrated activities | **Psychosocial support** |
|  | **Allied therapies** |
|  | **Health education** |
|  | **Spiritual or holistic practice** |
|  | **Socially-engaged practice** |
| Engagement | |
| Sensory stimuli | **Vision (sight)** |
|  | **Auditory (hearing)** |
|  | **Olfactory (smell)** |
|  | **Gustatory (taste)** |
|  | **Tactile (touch)** |
| Cognitive and/or creative stimuli | **Involvement of the imagination** |
|  | **Emotional stimuli** |
|  | **Cognitive stimuli** |
|  | **Aesthetic engagement** |
|  | **Pleasure** |
|  | **Participant choice** |
| Physical motions and actions | **Proprioception (kinaesthesia)** |
|  | **Movement** |
|  | **Physical exercises** |

| **PEOPLE** | |
| --- | --- |
| **Social composition** | |
| **Social diversity** | **Presence of others** |
|  | **Shared attributes** |
|  | **Distinct attributes** |
|  | **Personal attributes** |
| **Participant experience** | **Activity experience** |
|  | **Health experience** |
|  | **Lived experience** |
|  | **Relationship to others** |
| **Integrated social exchanges** | **Shared focus** |
|  | **Shared activity** |
|  | **Social exchanges** |
|  | **Structured social time during activity** |
|  | **Structured social time outside activity** |
|  | **Communications** |
| **Informal social exchanges** | **Unstructured social time during activity** |
|  | **Unstructured social time outside activity** |
| **Activity facilitation** | |
| **Type** | **Facilitator(s)** |
|  | **Co-production** |
|  | **Number** |
|  | **Professionalisation** |
|  | **Training** |
|  | **Consistency** |
| **Facilitator experience** | **Activity experience** |
|  | **Health experience** |
|  | **Lived experiences** |
|  | **Relationship to others** |
| **Practice and style** | **Technique** |
|  | **Personal attributes** |
|  | **Values-directed focus** |
|  | **Outcomes-directed focus** |
|  | **Person-centred focus** |
|  | **Autonomy-directed** |
|  | **Equality, diversity, and inclusion** |
|  | **Safety** |
|  | **Tailoring** |
| **Additional people** | **Presence of volunteers** |
|  | **Presence of healthcare professionals** |
|  | **Presence of others** |
| **CONTEXTS** | |
| **Setting** | |
| **Environment** | **Location** |
|  | **Basic features** |
|  | **Attractiveness** |
|  | **Situation** |
|  | **Time and day** |
|  | **Access** |
|  | **Privacy** |
| **Atmosphere** | **Comfort** |
|  | **Belonging** |
|  | **Familiarity** |
|  | **Ambiance** |
|  | **Organisation** |
| **Project set-up** | |
| **Economic resources** | **Participant charges** |
|  | **Project funding** |
|  | **Fees** |
|  | **Longevity** |
|  | **Environmental sustainability** |
| **Management** | **People** |
|  | **Affiliation** |
|  | **Branding** |
|  | **Collaboration** |
|  | **Patient and Public Involvement** |
| **Recruitment** | **Formal referral** |
|  | **Informal referral** |
|  | **Choice** |
|  | **Advertising** |
| **Signposting and referral** | **Inter-sector signposting** |
|  | **Health-sector signposting** |
|  | **Social signposting** |
|  | **Other-sector signposting** |
|  | **Safeguarding referral** |
